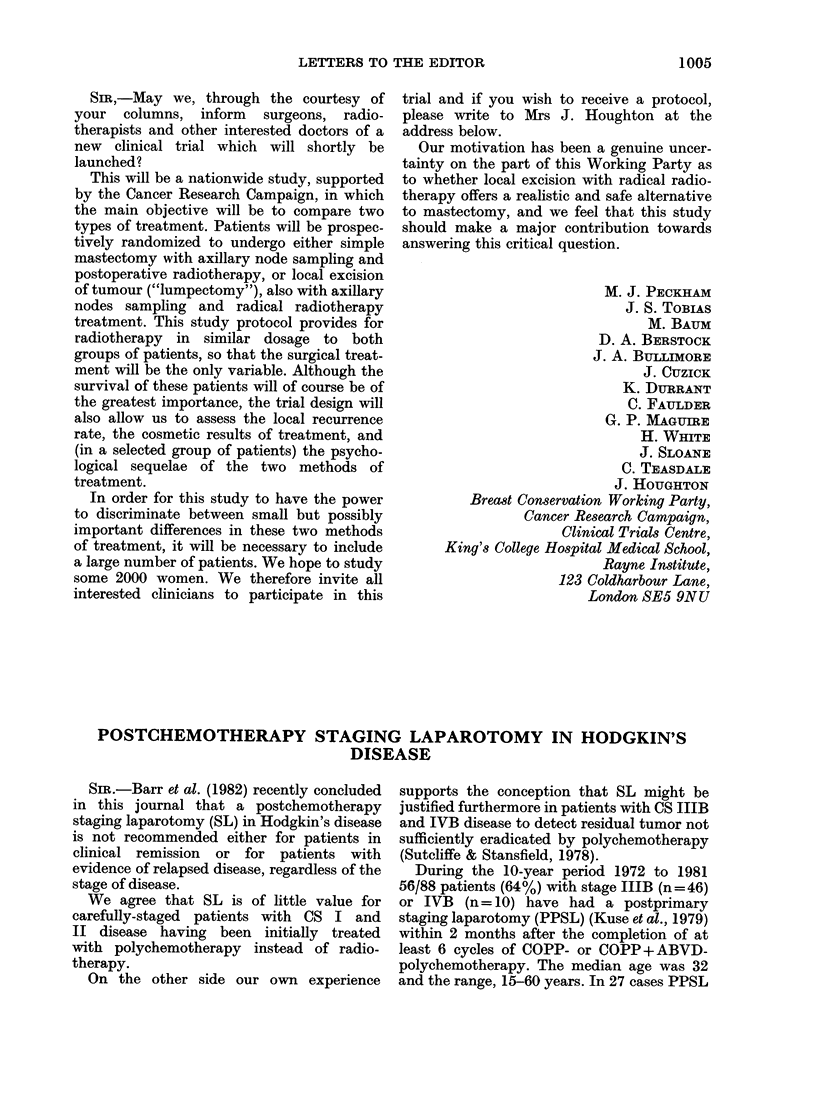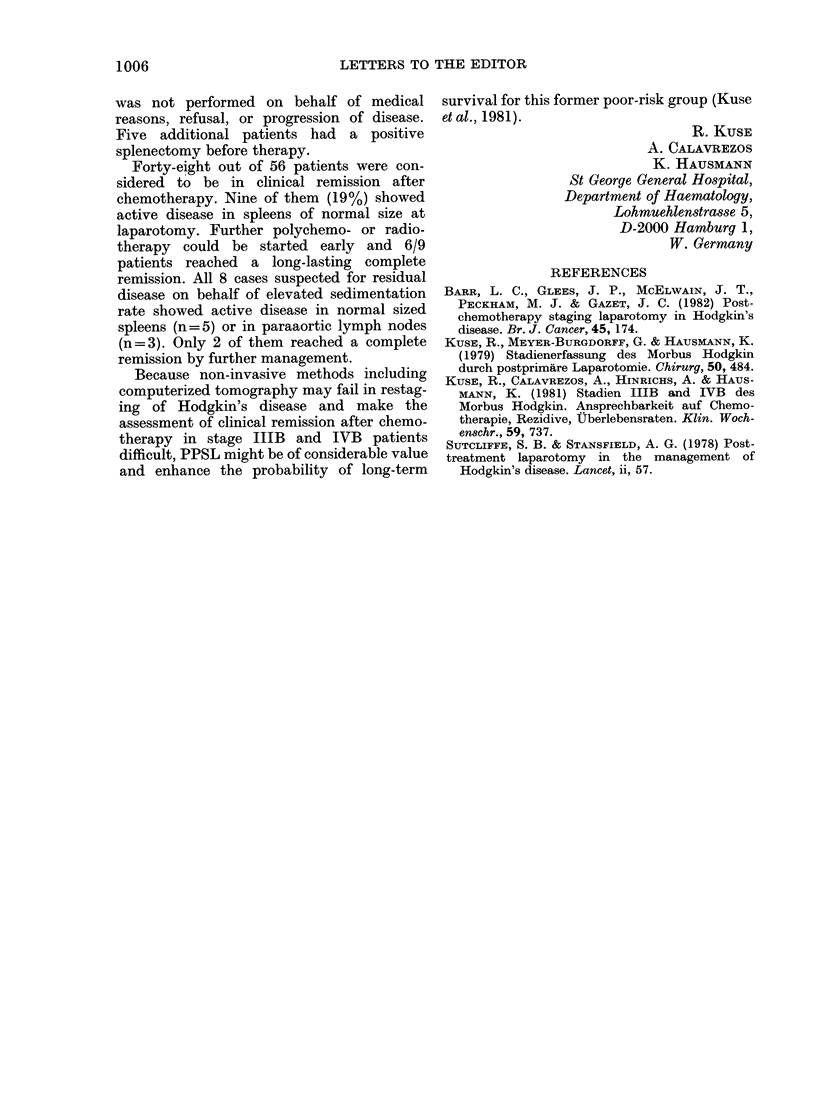# Postchemotherapy staging laparotomy in Hodgkin's disease.

**DOI:** 10.1038/bjc.1982.320

**Published:** 1982-12

**Authors:** R. Kuse, A. Calavrezos, K. Hausmann


### POSTCHEMOTHERAPY STAGING LAPAROTOMY IN HODGKIN'S DISEASE

SIR.—Barr *et al.* (1982) recently concluded in this journal that a postchemotherapy staging laparotomy (SL) in Hodgkin's disease is not recommended either for patients in clinical remission or for patients with evidence of relapsed disease, regardless of the stage of disease.

We agree that SL is of little value for carefully-staged patients with CS I and II disease having been initially treated with polychemotherapy instead of radiotherapy.

On the other side our own experience supports the conception that SL might be justified furthermore in patients with CS IIIB and IVB disease to detect residual tumor not sufficiently eradicated by polychemotherapy (Sutcliffe & Stansfield, 1978).

During the 10-year period 1972 to 1981 56/88 patients (64%) with stage IIIB (n = 46) or IVB (n = 10) have had a postprimary staging laparotomy (PPSL) (Kuse *et al.*, 1979) within 2 months after the completion of at least 6 cycles of COPP- or COPP + ABVD-polychemotherapy. The median age was 32 and the range, 15–60 years. In 27 cases PPSL

was not performed on behalf of medical reasons, refusal, or progression of disease. Five additional patients had a positive splenectomy before therapy.

Forty-eight out of 56 patients were considered to be in clinical remission after chemotherapy. Nine of them (19%) showed active disease in spleens of normal size at laparotomy. Further polychemo- or radiotherapy could be started early and 6/9 patients reached a long-lasting complete remission. All 8 cases suspected for residual disease on behalf of elevated sedimentation rate showed active disease in normal sized spleens (n=5) or in paraaortic lymph nodes (n=3). Only 2 of them reached a complete remission by further management.

Because non-invasive methods including computerized tomography may fail in restaging of Hodgkin's disease and make the assessment of clinical remission after chemotherapy in stage IIIB and IVB patients difficult, PPSL might be of considerable value and enhance the probability of long-term survival for this former poor-risk group (Kuse *et al.*, 1981).

R. Kuse
A. Calavrezos
K. Hausmann
*St George General Hospital,*
*Department of Haematology,*
*Lohmuehlenstrasse* 5,
*D*-2000 *Hamburg* 1,
*W. Germany*

REFERENCES

Barr, L. C., Glees, J. P., McElwain, J. T., Peckham, M. J. & Gazet, J. C. (1982) Post-chemotherapy staging laparotomy in Hodgkin's disease. *Br. J. Cancer*, **45**, 174.

Kuse, R., Meyer-Burgdorff, G. & Hausmann, K. (1979) Stadienerfassung des Morbus Hodgkin durch postprimäre Laparotomie. *Chirurg*, **50**, 484.

Kuse, R., Calavrezos, A., Hinrichs, A. & Hausmann, K. (1981) Stadien IIIB and IVB des Morbus Hodgkin. Ansprechbarkeit auf Chemotherapie, Rezidive, Überlebensraten. *Klin. Wochenschr.*, **59**, 737.

Sutcliffe, S. B. & Stansfield, A. G. (1978) Post-treatment laparotomy in the management of Hodgkin's disease. *Lancet*, ii, 57.